# Alternative materials for antegrade implantation of a double-J catheter in a public health system

**DOI:** 10.31744/einstein_journal/2023CE0437

**Published:** 2023-04-18

**Authors:** Guilherme Moratti Gilberto, Priscila Mina Falsarella, Luis Ricardo Socolowski, Arthur Munhoz Costa, Arthur Cesar de Souza Perin, Rodrigo Gobbo Garcia

**Affiliations:** 1 Hospital Municipal da Vila Santa Catarina Dr. Gilson de Cássia Marques de Carvalho Hospital Israelita Albert Einstein São Paulo SP Brazil Hospital Municipal da Vila Santa Catarina Dr. Gilson de Cássia Marques de Carvalho; Hospital Israelita Albert Einstein, São Paulo, SP, Brazil.; 2 Faculdade Israelita de Ciências da Saúde Albert Einstein Hospital Israelita Albert Einstein São Paulo SP Brazil Faculdade Israelita de Ciências da Saúde Albert Einstein, Hospital Israelita Albert Einstein, São Paulo, SP, Brazil.; 3 Hospital Israelita Albert Einstein São Paulo SP Brazil Hospital Israelita Albert Einstein, São Paulo, São Paulo, SP, Brazil.

Dear Editor,

Radioscopy-guided antegrade percutaneous implantation of a double-J catheter may be an alternative to retrograde insertion via cystoscopy in cases of neoplastic obstruction of the bladder trigone or anatomical distortions of the lower urinary tract that prevent retrograde catheterization.^(1,2)^ It is an alternative to nephrostomy, which can be performed simultaneously or afterwards, offering lower risks of infection, accidental loss of nephrostomy, and increased patient’s quality of life.^(3,4)^

The most widespread technique is based on Seldinger catheterization of the collecting system.^(3)^ Percutaneous access was performed using a Chiba needle under ultrasound guidance, followed by pyelography, which allowed for the insertion of a short 6 Fr sheath over a 0.035” Teflon J-tip guidewire. Transposition of the stenosing/obstructive lesion was performed using a hydrophilic guidewire. In the conventional technique, a 6 Fr x 45cm introducer sheath is used up to the bladder to support the progression of the double-J catheter.^(3)^

**Figure 1:**
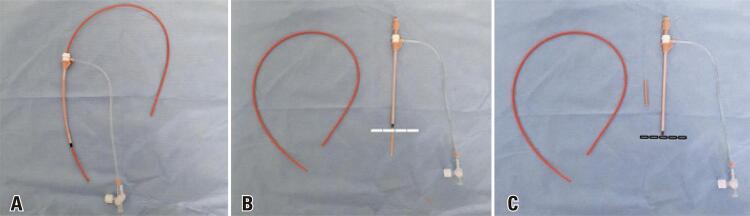
A) Short brite tip 7 Fr femoral sheath with double-J pusher; B) Short brite tip 7 Fr femoral sheath; C) Short brite tip 7 Fr femoral sheath with the dilator sectioned so that it has the same length as the introducer

**Figure 2:**
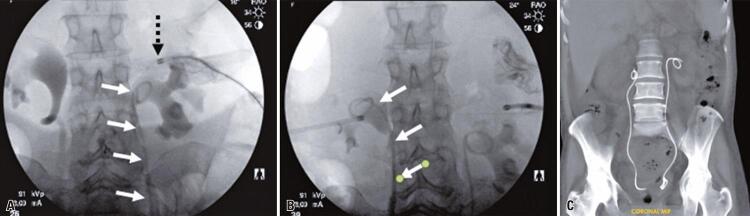
A) Pyelogram with the short brite tip 7 Fr femoral sheath used and placement of the double-J catheter into the left kidney (double-J catheter - white arrows; introducer radiopaque tip, black dashed arrow); B) Pyelogram with the short brite tip 7 Fr femoral sheath used and placement of the double-J catheter into the right kidney (double-J catheter - white arrows); C) Coronal tomography showing the final positioning of the double-J catheter

As a lower-cost alternative technique, we replaced the long sheath with a short brite tip 7 Fr femoral sheath. The double-J catheter was introduced through the short introducer sheath over Teflon or Amplatz guidewire under direct radioscopy visualization to guide the distal end into the bladder. Usually, these catheters are equipped with a pusher without a radiopaque tip, making it difficult to properly position its proximal end into the renal pelvis. Therefore, we chose to use an introducer dilator as the pusher. To achieve this, the dilator was sectioned such that it had the same length as the introducer (Figure 1). This ensured maximum progression of the proximal portion of the catheter into the renal pelvis, thus avoiding low positioning (Figure 2).

The major limitation of this method is the difficulty of the short sheath progression into the renal pelvis in patients with obesity.

In conclusion, use of a short sheath is a viable technique for anterograde drainage. Further studies are needed to compare the safety of this technique modification and its cost-effectiveness.
